# Zwitterionic Acetylated Cellulose Nanofibrils

**DOI:** 10.3390/molecules24173147

**Published:** 2019-08-29

**Authors:** Jowan Rostami, Aji P. Mathew, Ulrica Edlund

**Affiliations:** 1 Fiber and Polymer Technology, KTH Royal Institute of Technology, SE-10044 Stockholm, Sweden; 2 Department of Materials and Environmental Chemistry, Stockholm University, SE-10691 Stockholm, Sweden

**Keywords:** cellulose nanofibrils, zwitterionic, acetylation, CNF

## Abstract

A strategy is devised to synthesize zwitterionic acetylated cellulose nanofibrils (CNF). The strategy included acetylation, periodate oxidation, Schiff base reaction, borohydride reduction, and a quaternary ammonium reaction. Acetylation was performed in glacial acetic acid with a short reaction time of 90 min, yielding, on average, mono-acetylated CNF with hydroxyl groups available for further modification. The products from each step were characterized by FTIR spectroscopy, ζ-potential, SEM-EDS, AFM, and titration to track and verify the structural changes along the sequential modification route.

## 1. Introduction

The scope of application of nanocelluloses, including cellulose nanofibrils (CNF), microfibrillated cellulose (MFC), and cellulose nanocrystals (CNCs) [1], may be significantly extended by the engineering of the surface chemistry. Nanocellulose has many good features and stands out as a very promising feedstock for creating advanced bio-based materials that can compete with conventional ones, but there also challenges [2,3,4,5,6]. Limiting properties, such as dimensional instability in humid conditions, restricted solubility, lack of thermoplasticity, limited compatibility with composite matrices, and hornification upon drying, are due to the abundance of hydroxyl groups and, hence, strong hydrogen interactions and high hydrophilicity. Also, susceptibility to microbial attack and biofouling may limit the applicability of nanocellulose materials in various applications.

Chemical functionalization is a way forward. The cellulose molecule can be modified in many ways, primarily by reactions with hydroxyl groups, the reducing end, or by ring-opening of the main chain d-anhydroglucopyranose units (AGUs). Covalent modification of nanocellulose, including oxidation, esterification, etherification, grafting copolymerization, and ring-opening reactions, has recently been the subject of several comprehensive reviews [3,6,7]. Some surface modifications have been directly translated to nanocellulose from known and industrially implemented cellulose pulp reactions, not in the least esterification and etherification [8]. Esterification, acetylation being a well-known example, typically proceeds via base activation of the hydroxyl groups followed by reaction with an acyl halide, anhydride, or carboxylic acid. Cellulose acetate, CA, was first synthesized with acetic anhydride as the esterification agent [9]. Since then, a range of routes has been demonstrated in which CA is prepared with different degrees of substitution (DS) and distribution of substituents [10,11]. Controlling DS is always a challenge, yet a limited DS provides opportunities to alter properties (e.g., hydrophilicity) [12] while keeping remaining hydroxyl group(s) in each AGU available for subsequent modification. In that sense, cellulose acetylation finds use as a pre-treatment strategy [13] to overcome the limitations in solubility of nanocellulose and allow for chemical functionalization pathways which run efficiently in greener solvents.

Functionalization of nanocelluloses with ionic groups may be useful for many purposes. In biomedical applications, an- and cat-ionized nanocelluloses may serve as pH-sensitive hydrogels, or stimulate cell proliferation due to electrostatic interactions [14,15]. Sulfonated CNF was prepared to decrease pro-coagulant activity and investigated as a potential hemocompatible biomaterial [16]. Ionically functionalized CNF was also shown to have antimicrobial properties, with death rates of 98% or even higher for *Candida oleophila* [17]. In water purification, ionic nanocellulose may provide an enhanced ability to capture charged molecules and heavy metal ions from contaminated effluents [18], for instance, cationic CNF with quaternary ammonium groups, and proved their usefulness in water treatment processes as they could retain fluoride, nitrate, and other anions [19].

A zwitterionic macromolecule adds another dimension of functionality and applicability of nanocellulose materials. Zwitterionic polymers typically contain the same number of positive and negative ionic charges, and the overall charge is zero under normal conditions despite the high density of ion pairs attached to the polymer chains [20]. Zwitterionic polymers have been investigated for their ability to prevent biofouling in various applications. Polymeric zwitterionic membranes were shown to effectively reduce nonspecific protein adsorption, bacterial adhesion, and (bio)fouling [21,22]. A few attempts to turn nanocelluloses zwitterionic have been reported in the recent literature. Edler et al. grafted glycidyltrimethylammonium chloride onto TEMPO/NaBr/NaOCl-oxidized CNF. The final modified CNF responded to ionic surfactants with controlled self-assembly [23]. Liu et al. used acetylated cellulose to prepare a zwitterionic ultrafiltration membrane material. The modification afforded increased hydrophilicity, antifouling properties, enlarged the pore sizes, and amplified the porosity, which in turn led to augmented water flux [24]. Antifouling and multimodal electrostatic interactions are properties that render zwitterionic nanocelluloses highly interesting for a number of potential applications. Our aim was to devise a new route—controllable and sequential—for the production of zwitterionic CNF. The procedure is inspired by the pathway brought forward by Liu et al. [24], however CNF is used as the starting material, acetylation is added to the procedure, and the following periodate oxidation protocol was improved.

## 2. Results

**Scheme 1:**
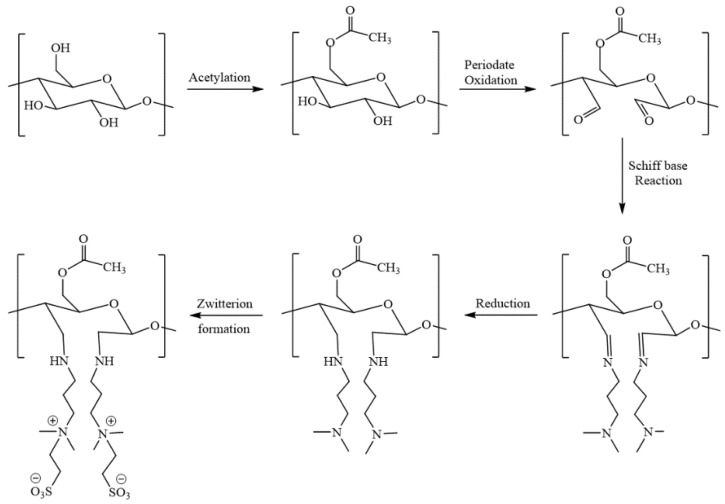
Sequential synthesis of zwitterionic acetylated cellulose nanofibrils (CNF).

CNF were modified via acetylation, periodate oxidation, Schiff base formation, and reduction, as well as a quaternary ammonium reaction, to achieve zwitterionic properties (Scheme 1). Special attention was put in to modify current protocols for acetylation and periodate oxidation steps to reduce reaction times [24]. The products from each step were characterized with FTIR spectroscopy, ζ-potential, SEM-EDS, AFM, and titration to track and verify the structural changes along the sequential modification route. Images from SEM and AFM morphological analyses of the wood-derived CNF and the produced zwitterionic and acetylated CNF are available in the supplementary material (Figures S1 and S2, Supplementary Material).

### 2.1. Acetylated CNF (ACNF)

ACNF were synthesized with acetic anhydride in the presence of acetic acid and catalytic amounts of sulfuric acid (Scheme 1). Sulfuric acid is a commonly applied catalyst as it will rapidly bind to the surface of cellulose which then proceeds to the formation of acetyl groups by reaction with acetic anhydride [25,26]. Acid-catalyzed hydrolysis can occur if water is present in the system [26], affecting the yield, and hence acetylation is often performed in other media, including dimethylacetamide (DMAc)/lithium chloride, which is not optimal for reasons of cost and sustainability. Glacial acetic acid presents an alternative medium but due to the long reaction times applied, there is a risk of concurrent cellulose de-polymerization [27]. We used glacial acetic acid as the reaction medium and reduced the reaction time to 15 min. The reaction was performed at room temperature. The acetylation reaction was purposely driven to a fairly low degree of substitution to alter the hydrophilicity while still maintaining the majority of the OH groups for future modifications. 

**Figure 1:**
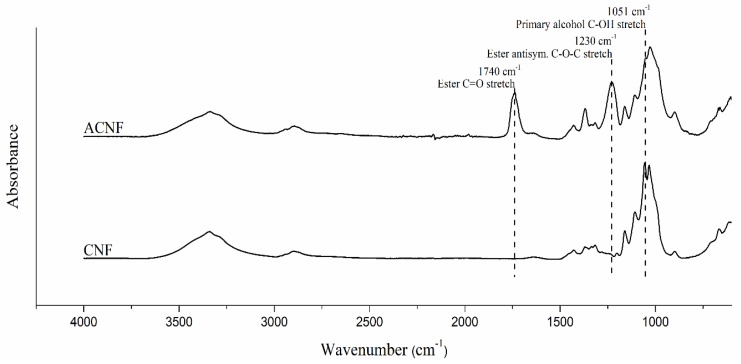
ATR-FTIR spectra of CNF and acetylated CNF (ACNF).

The successful and partial acetylation of CNF was confirmed with FTIR spectroscopy (Figure 1). A carbonyl C=O stretch at 1740 cm^−1^ and an anti-symmetric stretch C–O–C stretch at 1230 cm^−1^, characteristic of ester pendant groups, were detected in ACNF but not in CNF. The average extent of acetylation was estimated from the FTIR spectrum of ACNF by calculating the ratio (r) between the absorbance of the C=O present in ester groups (1740 cm^−1^) and the absorbance of the C–O present in the cellulose backbone (1030 cm^−1^) according to Equation (1) where r denotes the degree of modification [28]:

(1) $$r=\frac{A_{C=O\;\left(ester\;group\right)}}{A_{C-O\;\left(cellulose\;backbone\right)}}.$$

The extent of acetylation was estimated to ca. 30% with Equation (1), meaning that the produced ACNF is essentially mono-acetylated. A more precise determination of the number of acetyl groups was done by titration according to Equation (2) (Section 4.7.5.). The extent of acetylation was thereby quantified as 30 mol acetyl groups/mol ACNF, i.e., the extent of acetylation was 30%. The acetylation reaction is not selective, still, the primary hydroxyl group is expected to be more reactive than the secondary OH groups for steric reasons. FTIR spectra sustain this assumption, showing a significant decrease in the primary alcohol band at 1051 cm^−1^ for ACNF when compared to CNF. 

The use of sulfuric acid as a catalyst, albeit at a minute amount, is a possible concern if sulfate groups, formed as intermediates in the reaction, remain in the final product. Elemental composition analysis with SEM-EDS showed that ACNF do not contain any detectable traces of sulfur. 

The ζ-potential value serves as an indicator for the charges present at the surface of suspended particles and/or be an indicator for the stability of colloids in the suspension [29]. A ζ-potential value in the 0 to ±5 mV range corresponds to flocculation and rapid coagulation, i.e., the suspension is highly unstable. A value in the range of ±10 to ±30 mV indicates the emerging instability of the colloid. Any value beyond ±30 mV can be interpreted as pronounced stability for the colloids in the suspension as they coagulate less and disperse more easily [29]. The ζ-potential value of ACNF was significantly lower than the ζ-potential of the starting material and approached the stable region of −30 mV (Table 1), further sustaining a successful acetylation reaction. 

### 2.2. Oxidized Acetylated CNF

Periodate oxidation stands out as a viable pathway to oxidized cellulose given the mild conditions at which the reaction can be carried out, e.g., at room temperature. The reaction is highly precise and rarely leads to any side reactions [30]. The generated aldehyde groups are highly reactive and can, in turn, be utilized for further modifications. Here, ACNF were oxidized with sodium(meta)periodate. Two alternative routes were explored: Oxidation in DMSO and oxidation in water. The latter was performed with a shorter reaction time than in the literature. From a green chemistry perspective, water is the better choice and when preliminary results showed that satisfactory results were obtained in water, this route was chosen for further studies. The proposed mechanism for periodate oxidation includes the reaction of the periodate anion with the vicinal OH groups at C2 and C3 positions, generating a cyclic iodate diester intermediate. A ring-opening reaction then occurs, forming dialdehyde cellulose and iodate (IO_3_^−^) ions (Scheme S1, Supplementary Material) [31,32]. Acetylation of the primary alcohols will not significantly disturb the oxidation reaction as only vicinal OH groups are involved in the periodate oxidation. 

**Figure 2:**
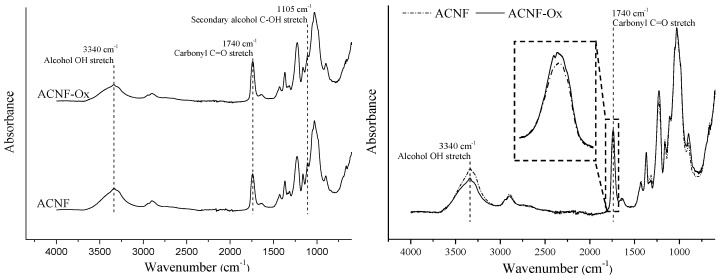
FTIR spectra of ACNF and oxidized ACNF (ACNF-Ox): Stacked (**left**) and overlaid (**right**).

FTIR spectra of ACNF and oxidized ACNF (ACNF-Ox) are quite similar (Figure 2). Acetylation introduced aliphatic esters to the structure, reflected in a significant carbonyl stretch band at 1740 cm^−1^. Since aldehydes introduced by periodate oxidation also contain C=O, a band at approximately the same wavenumber is expected for ACNF-Ox. When the spectra are baseline corrected and normalized, however, the relevant peaks in both samples can be compared based on the energy absorbed at the different regions. A clear decrease in absorption is then observed for the OH band around 3340 cm^−1^ for ACNF-Ox compared to ACNF, while the intensity of the carbonyl band is increased. This indicates that some of the secondary OH groups have undergone oxidation to aldehyde groups. This is further sustained by a decrease in intensity of the secondary alcohol band at 1105 cm^−1^ for ACNF-Ox (Figure 2). 

ACNF-Ox were titrated with hydroxylamine hydrochloride and the average number of aldehyde groups was quantified according to Equation (3). The calculated extent of oxidation was 34%. A linear polymer-like structure could possibly be obtained at certain regions of CNF as the fibril structure is lost due to ring-opening. The crystal structure gets increasingly disrupted as the rings open up due to a significant reduction in hydrogen bonds. In this work, we targeted a limited degree of aldehyde content in order to preserve the nanofibrillar and crystal structure of CNF as much as possible.

The ζ-potential of ACNF-Ox was −33.7 ± 0.23 mV (Table 1), which is somewhat lower than the ζ-potential values for ACNF and CNF, respectively. The decrease in ζ-potential further sustains the change in structure brought about by the oxidation reaction and can be explained by a reduction of hydrogen bonds as OH groups have oxidized to aldehydes. Hence, the chains become less densely packed, coagulate less, and become more stable in the suspension. 

### 2.3. Aminated Acetylated CNF

**Scheme 2:**
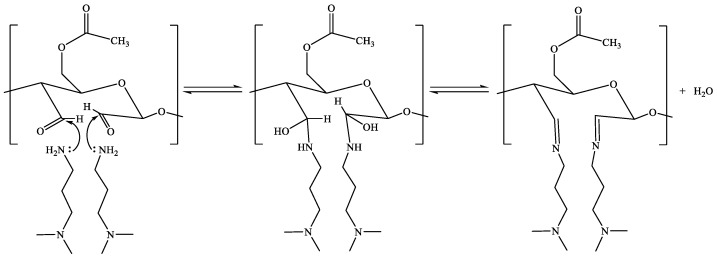
Proposed mechanism for the formation of Schiff base groups from ACNF.

Aminated ACNF (ACNF-Am) were synthesized from ACNF-Ox and *N*,*N*-dimethyl-1,3 propanediamine (Scheme 2). The primary amine will act as a nucleophile and perform a nucleophilic attack on the electrophilic aldehyde groups present in ACNF-Ox. This leads to the formation of a highly unstable intermediate: An alcohol carbinolamine. The cleavage of a water molecule leads to the formation of Schiff base groups. The alcohol carbinolamine will exist in equilibrium with the Schiff bases as the reaction is subjected to an aqueous medium and water is formed as a by-product [33]. Both steps of the Schiff base formation are reversible and occur both under basic and acidic conditions. The dehydration of the alcohol carbinolamine intermediate is rate-determining; thus, it is of outmost importance that the reaction is catalyzed, for example by an acid, to drive the dehydration of the intermediate (Scheme S2, Supplementary Material). The acidic medium must be relatively weak due to the basic nature of amines [33]. If the acidic strength is too high, the formation of alcohol carbinolamine will not occur as the amines will become protonated and lose their nucleophilic properties [34]. We performed the Schiff base reaction in an acetate buffer with pH = 4.5. 

**Scheme 3:**
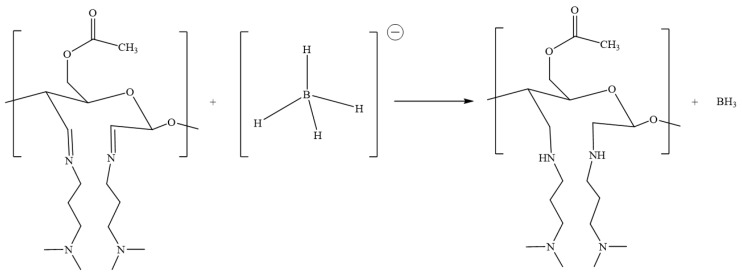
The sodium borohydride reduction of the Schiff base groups.

After completion of the Schiff base reaction (6 h), we added an aqueous sodium borohydride solution to the reaction mixture to reduce the double bond in the Schiff base groups to secondary amine groups, ACNF-Am (Scheme 3). This step was performed due to the instability and reactivity of Schiff bases. Particularly, there was a risk that further modifications would cause unwanted side reactions. 

**Figure 3:**
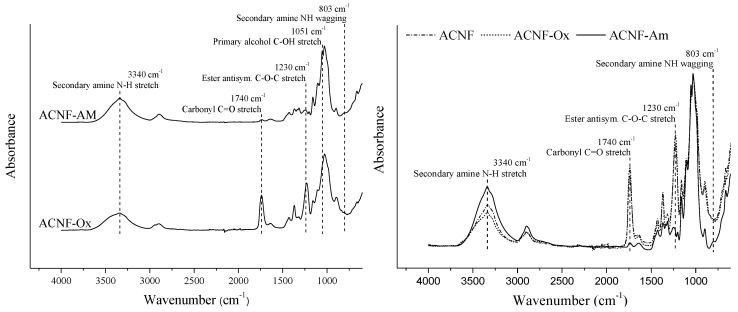
FTIR spectra of ACNF, ACNF-Ox and aminated ACNF (ACNF-Am): Stacked (**left**) and overlaid (**right**).

**Figure 4:**
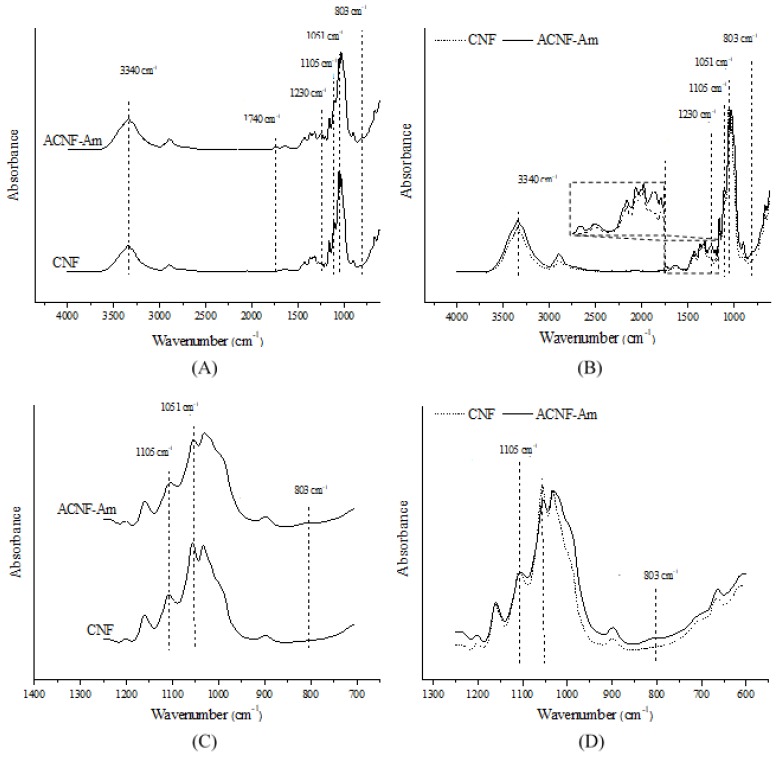
FTIR spectra of (**A**) CNF and ACNF-Am stacked, (**B**) CNF (dashed-dotted line) and ACNF-Am (solid line) overlaid, (**C**) spectral region 1250–700 cm^−1^ of (**A**), and (**D**) spectral region 1250–700 cm^−1^ of (**B**).

FTIR spectra sustain the formation of ACNF-Am (Figure 3). The representative NH stretch of secondary amines is usually detected in the region 3350–3300 cm^−1^. This band overlaps with the broad OH band in the 3340 cm^−1^ region, which makes it very difficult to distinguish one band from the other. When the spectra are baseline corrected and normalized, however, it is clear that there is a rather large increase in absorption intensity for ACNF-Am around 3340 cm^−1^, compared to ACNF and ACNF-Ox (Figure 3, left). NH wagging vibration in secondary amines is typically observed at 785–720 cm^−1^ but peaks may be slightly displaced in the fingerprint region and shifting can occur. Thus, the small peak detected at 803 cm^−1^ could be assigned to the NH wagging in the secondary amine (Figure 3 and Figure 4). This peak is not present in the FTIR spectra of CNF, ACNF, and ACNF-Ox (Figure 3 and Figure 4). No additional peak or shoulders are detected at 1690–1630 cm^−1^, where a C=N stretch band originating from Schiff base groups is expected. This indicated the successful conversion of Schiff base groups to secondary amine groups. The C–N stretch in tertiary aliphatic amines occurs in the 1240–1030 cm^−1^ region but cannot be detected due to significant overlap with other significantly larger bands already present in this region. The intensity of the carbonyl band at around 1740 cm^−1^ is significantly lower for ACNF-Am when compared to ACNF and ACNF-Ox (Figure 3), which indicates that aldehyde groups have successfully been converted to secondary amines. Still, the reduction in the intensity of the carbonyl band may also suggest that both aldehyde groups and acetyl groups have undergone reactions. Sodium borohydride is a relatively weak nucleophile and it is not likely to react with the acetyl ester groups as they are very stable compared to the aldehyde groups. The reduction of aliphatic ester groups occurs very slowly in protic solvents [35]. The acetyl groups may however partially hydrolyze to the corresponding alcohol groups, due to the acidic environment. Indeed, a decrease in intensity of the ester antisymmetric C–O–C stretch at 1230 cm^−1^ and an increase in the primary alcohol band at 1051 cm^−1^ is observed for ACNF-Am when compared to FTIR spectra of ACNF-Ox and ACNF-Am (Figure 3, right). The increase in intensity at 3340 cm^−1^ seems, therefore, to originate from both secondary amines and newly formed OH groups from the hydrolyzed acetyl groups. 

The FTIR spectra of pure CNF and ACNF-Am were compared to further investigate if amine groups were successfully synthesized and if the increase in intensity around 3340 cm^−1^ is not solely due to the newly formed OH groups (Figure 4). The primary alcohol band at 1051 cm^−1^ is less eminent for ACNF-Am than for CNF, indicating that primary alcohols were partially acetylated and remained acetylated (Figure 4C,D). ACNF-Am still gives rise to a small ester C=O band at 1740 cm^−1^ and an antisymmetric ester band at 1230 cm^−1^, peaks which are not in the spectrum of CNF. The secondary alcohol peak at 1105 cm^−1^ is less pronounced for ACNF-Am than for CNF, indicating that secondary OH groups were oxidized and not substituted back to OH groups (Figure 4C). Most importantly, the alcohol OH stretch and the secondary amine NH stretch both give rise to bands around 3340 cm^−1^. The amplified absorption at 3340 cm^−1^ observed for ACNF-Am, despite the reduction of OH groups due to, e.g., acetylation, verifies the presence of secondary amine groups. Otherwise, a decrease in intensity at 3340 cm^−1^ should have been observed for ACNF-Am. The Schiff base and reduction reactions were replicated several times and the results obtained by FTIR spectroscopy remained unchanged each time.

The ζ-potential of ACNF-Am was 1.5 ± 1.21 mV, which is markedly different from the value obtained for ACNF-Ox (Table 1). This large increase in ζ-potential may be explained by the basic nature of amines. Amine groups are easily protonated and may thus give rise to positive charges. The obtained positive ζ-potential value suggests the successful amination modification of ACNF-Ox to ACNF-Am. The standard deviation is rather large, and this is probably due to the instability of the particles in the suspension. The particles are likely to coagulate fast and sediment easily, which influences measurements over time. 

### 2.4. Zwitterionic Acetylated CNF

**Scheme 4:**
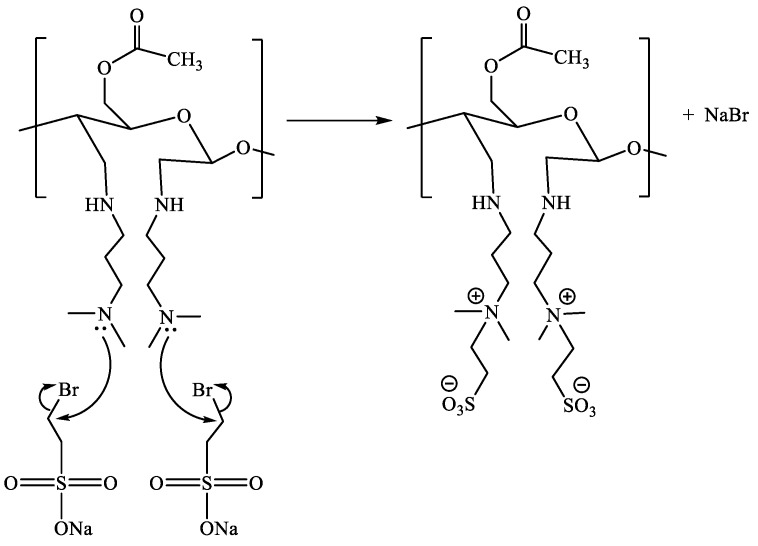
The reaction of ACNF-Am with sodium 2-bromoethanesulfonate (SBES), producing zwitterionic ACNF (ACNF-Zwi) and sodium bromide (NaBr).

Amines can undergo further reactions to form quaternary ammonium compounds. We synthesized zwitterionic ACNF (ACNF-Zwi) by reacting ACNF-Am with sodium 2-bromoethanesulfonate (SBES) (Scheme 4). The reaction is believed to occur at the site of the tertiary amine groups, which forms quaternary ammonium cations and sulfonate anions. Secondary amines are better nucleophiles than tertiary amines, however the secondary amines in ACNF-Am are significantly sterically hindered by the rest of the ring. Nevertheless, the probability that secondary amines have reacted with SBES cannot be excluded. 

**Figure 5:**
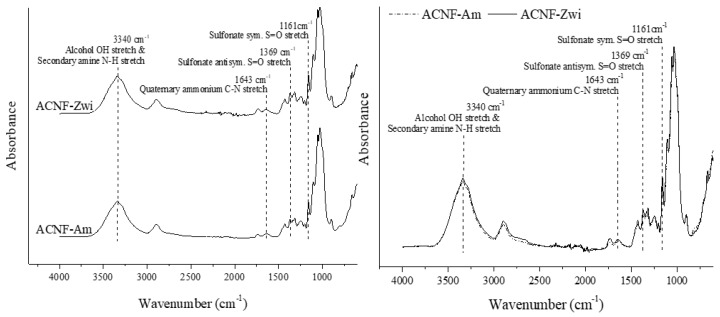
FTIR spectra of ACNF-Am and ACNF-Zwi: Stacked (**left**) and overlaid (**right**).

FTIR spectra of ACNF-Am and ACNF-Zwi are quite similar (Figure 5). The introduced groups, i.e., sulfonate anions and quaternary ammonium cations, are difficult to detect in the FTIR spectra. Sulfonate groups typically give rise to a symmetric S=O stretch band in the 1195–1168 cm^−1^ region, but due to overlap, this band is poorly resolved. Still, there is an increase in absorption at 1161 cm^−1^ which could be attributed to the sulfonate symmetric S=O stretch. An increase is also observed at 1369 cm^−1^, which could be assigned to the sulfonate antisymmetric S=O stretch. Weak quaternary ammonium N–C stretches can usually be detected at 1639–1645 cm^−1^ [24]. Water bound to the CNF structure gives rise to a band in this region in all samples and effectively masks other weak bands in the same region.

The elemental composition of ACNF-Zwi was determined with SEM-EDS to further investigate if the quaternary ammonium formation reaction was successful. If so, the presence of sulfur will indicate the presence of sulfonate groups in the structure. The SEM-EDS instrument we used does not accurately detect hydrogen and the atom numbers given for light elements (atom numbers < 10), such as oxygen, nitrogen, and carbon, are highly approximate. Still, sulfur is measurable and was indeed detected in ACNF-Zwi (1.5 ± 0.2%). An EDS spectrum is available in the supplementary material (Figure S3, Supplementary Material). Sulfur was not detected in CNF and ACNF, providing further indication that ACNF-Zwi contains immobilized sulfonate groups. The ζ-potential of ACNF-Zwi was −6.6 ± 0.17, which is somewhat lower than the ζ-potential of ACNF-Am (Table 1). A decrease in the ζ-potential indicates that negatively charged sulfonate groups were introduced to the structure. The change is not that significant, however the results indicate that positively charged quaternary ammonium cations are introduced to the structure, which counteracts the negative charges. 

SEM and AFM images of CNF and the produced ACNF and ACNF-Zwi are available in the Supplementary information (Figures S1 and S2). The nanofibers were aggregated to a great extent in all cases probably due to the freeze-drying process and, in the case of AFM, the sample preparation step involving drying on mica. Nanoscale fibers, as well as micron-scaled bundles on nanofibers, were visible in all samples. CNF and ACNF-Zwi were relatively easy to disperse in water while the ACNF did not re-disperse well in water even after sonication. This is expected due to the somewhat hydrophobic nature of acetylated CNF. This was also reflected in the sizes of nanofibers (measured using Nanoscope software), which varied between 22–54, 30–115, and 17–44 nm for CNF, A-CNF, and ZI-CNF, respectively.

## 3. Discussion

The sequential modification scheme for CNF described here was elaborated and investigated in an effort to broaden the library of CNF derivatives and potentially open up for new applications, where CNF is currently not considered a viable component. Nanocellulose has many interesting, and potentially valuable, properties and is often put forth as a high-performance component candidate in future, sustainable materials, not in the least thanks to their inherent renewability [2,3,4,5,6,7]. Still, renewability is not self-sufficient from a sustainability point of view [36]. Green process conditions are of vital importance and even small improvements, such as a reduction of the energy consumption, substitution of organic solvents with water, and/or the use of efficient catalysts, can have a significant impact on the overall environmental impact of the process. CNF modification and processing presents a challenge in this respect due to the insolubility in water and with very few sustainable solvent alternatives at hand [36]. Heterogeneous modification is often possible but with less control of yield, degree of substitution, selectivity, and by-products. 

Here we tried to take the above into account throughout the functionalization route. The first step, mono-acetylation, was introduced to alter the hydrophilicity. The reaction was performed in glacial acetic acid, considered a more eco-friendly solvent than many other solvents employed for acetylation of cellulose [26]. There is always a risk of partial degradation during acetylation: De-acetylation can occur due to acid-catalyzed hydrolysis and degradation may occur since the glycosidic oxygen present in the backbone of cellulose is susceptible to acidic hydrolysis. We reduced the reaction time to 15 min to suppress the exposure time of CNF to the acidic environment. Moreover, the reaction was carried out at room temperature, as the reaction between acetic anhydride and cellulose is exothermic and a lower temperature decreases the degradation degree. Ice-cold water was used throughout the acetylation process for the same reason: For terminating the reaction, washing and for dialysis.

The first protocol for periodate oxidation of ACNF used DMSO as a medium, nitrogen as an atmosphere and allowed the reaction to occur for 6 h at 45 °C [24]. The protocol was improved by changing the medium to DI water and the atmosphere from nitrogen to air. Furthermore, the reaction was carried out at room temperature and the reaction time was decreased to 90 min. The obtained degree of oxidation corresponded to 34%. 

The final step most likely produced zwitterionic CNF with quaternary ammonium groups. Such functionalities open up new avenues of possible applications of CNF. Quaternary ammonium cations are permanently charged species at a variety of different pH values and are unreactive toward oxidants, acids, electrophiles, and nucleophiles. The hydrophilic structure of ACNF-Zwi could be beneficial for future use in water filter systems as the membranes are more easily hydrated which could simplify interactions between the zwitterionic groups and heavy metal contaminants in water effluents [37]. As quaternary ammonium cations are charged at a vast spectrum of pH values, the use of these compounds in filter membranes could facilitate the purification of water in different conditions. Other potential applications include surface-active agents, antimicrobials, non-fouling surfaces, and surfactants.

## 4. Materials and Methods

### 4.1. Materials

Sulfuric acid 95–98%, acetone, sodium (meta) periodate, ethylene glycol ≥99%, *N*,*N*-dimethyl-1,3-propanediamine 99%, sodium borohydride, sodium acetate ≥99% and sodium 2-bromoethanesulfonate 98% (SBES) and hydroxylamine hydrochloride ≥99.9995% were purchased from Sigma-Aldrich (Stockholm, Sweden). Acetic anhydride ≥99.0% was purchased from Fluka Chemika (distributed by Fischer Scientific, Gothenburg, Sweden). Glacial acetic acid 99.79% was purchased from Fisher Scientific (Gothenburg, Sweden). Dimethyl sulfoxide ≥99% (DMSO) was purchased from Merck (Stockholm, Sweden). Hydrochloric acid (HCl) was purchased from J.T. Baker (distributed via VWR, Stockholm, Sweden). Absolute ethanol and sodium hydroxide (NaOH) 99.3% were purchased from VWR Chemicals (Stockholm, Sweden). Microfibrillated cellulose paste (Exilva P01-V) with a solid content of 10.1% (w/w) was a kind gift from Borregaard (Sarpsburg, Norway). Exilva P01-V has a viscosity (2% in H_2_O) of 23,000 mPas and a conductivity (2% in H_2_O) of 23 μS/cm. Deionized (DI) water was used throughout the study. All chemicals were used without further purification.

### 4.2. Lyophilization

The CNF paste (35 g, 10.1% w/w) was suspended in DI water (200 mL) in a round-bottomed flask (500 mL) and stirred until the nanofibers were thoroughly dispersed in the aqueous phase. Then, the suspension was probe sonicated (10 min, pulse on/off for 10 s, respectively) using a CV334 probe sonicator from CIAB Chemical instruments AB with a maximum input power of 750 W and a maximum frequency of 20,000 times/s at an amplitude of 20%. Afterward, the suspension was put in liquid nitrogen, under constant rotation, until the mixture became completely frozen. The frozen suspensions were then lyophilized using a SCANVAC CoolSafe freeze dryer with a vacuum of 0.250 hPa and a condenser temperature of −82 °C for 72 h.

### 4.3. Acetylation of CNF (ACNF)

The acetylation reaction was developed by modification of a previously described protocol [28] to reduce the reaction time. Freeze-dried CNF (5 g) was added successively to glacial acetic acid (125 mL) under stirring. Acetic anhydride (25 mL) was added to the mixture, followed by the addition of sulfuric acid (50 μL). The mixture was left to react under vigorous stirring and at room temperature for 15 min before the reaction was terminated by the addition of a large excess of ice-cold DI water. The mixture was then centrifuged (4000 rpm, 20 min), using an Avanti J-E centrifuge from Beckman Coulter (Carlsbad, CA, USA). The supernatants were removed and replaced with new ice-cold DI water and centrifuged again. The centrifugation and re-dispersion procedure was repeated 8 times before the product was obtained by collecting the pellet. The product was transferred to a Spectra/Por dialysis membrane, with a molecular weight cut-off (MWCO) of 6000–8000 g/mol, and dialyzed for 7 days. The DI water used throughout the dialysis process was exchanged with fresh ice-cold DI water 2–3 times each day. Lastly, the product was collected from the dialysis membranes and the moisture content of the acetylated CNF (ACNF) was determined by drying a known amount of product in an oven at 60 °C for 24 h. The obtained ACNF was kept at 4 °C until further use.

### 4.4. Oxidation of Acetylated CNF (ACNF-Ox)

Periodate oxidation was explored via two different protocols: One performed in DMSO and one using water as the reaction medium. 

#### 4.4.1. Oxidation in DMSO

ACNF suspension was solvent exchanged from water to DMSO according to a procedure described elsewhere [38]. Briefly, ACNF (7 g) was suspended in DI water (50 mL) and stirred for 5 h. The mixture was then probe sonicated (5 min, pulse on/off for 10 s, respectively), using a CV334 probe sonicator from CIAB Chemical instruments AB with a maximum input power of 750 W, and a maximum frequency of 20,000 times/s at an amplitude of 20%, before the suspension was again stirred for 3 h. The mixture was then distributed to centrifugation tubes (10 mL per tube), followed by the addition of DMSO (30 mL) to each tube. The suspensions were then centrifuged (4000 rpm, 20 min), using an Avanti J-E centrifuge from Beckman Coulter. The supernatants were removed and replaced with fresh DMSO and centrifuged again. The centrifugation/re-dispersion cycle was repeated 4 times, before collecting the pellets. The DMSO content in each ACNF pellet was determined through pre-weighting of the samples as well as by oven drying test.

The oxidation was then carried out in DMSO and in the dark [24]. The solvent exchanged ACNF (1 g, dry weight) was added to a round bottomed flask containing DMSO (20 mL). Sodium (meta) periodate (0.5 g) was added to the reaction flask in the dark. The pH of the reaction mixture was adjusted to 3.5 with sulfuric acid (1 M). The reaction was then allowed to proceed for 6 h at 45 °C under nitrogen atmosphere, before it was terminated by the addition of ethylene glycol and washed with DI water through centrifugation and re-dispersion.

#### 4.4.2. Oxidation in Water

A previous protocol for periodate oxidation [39] was modified slightly to reduce the reaction time. ACNF (1 g, dry weight) was suspended in an aqueous solution (250 mL) containing 6.3% (w/w) 2-propanol, followed by the addition of sodium (meta) periodate (5.4 g). The role of 2-propanol is to serve as a radical scavenger. The mixture was left to react for 90 min at 45 °C in the dark. The reaction was terminated by the addition of ethylene glycol and washed with DI water through centrifugation and re-dispersion.

### 4.5. CNF Schiff Base Formation and Reduction (ACNF-Am)

ACNF-Ox (0.5 g, dry weight) was dispersed in acetate buffer (25 mL, pH 4.5, prepared from 400 mL of sodium acetate (0.2 M) mixed with 500 mL of acetic acid (0.2 M)) according to a previous protocol [24]. The mixture was stirred for 15 min before *N,N*-dimethyl-1,3-propanediamine (3 equivalents with respect to ACNF-Ox) was added under a nitrogen atmosphere. The reaction mixture was heated to 45 °C and the mixture was left to react for 6 h. The reaction slowly turned a faint yellow color which turned darker with time. Next, 2 equivalents (with respect to ACNF) of 5% (w/v) sodium borohydride aqueous solution were added slowly to the mixture through a syringe. The mixture was left to react, under the same conditions, for an additional 3 h. The reaction was terminated by the addition of a large excess of ice-cold DI water. The mixture was washed by centrifugation (4000 rpm, 20 min), using an Avanti J-E centrifuge from Beckman Coulter, and re-dispersion with ice-cold DI water. This washing procedure was repeated 8 times before the product was acquired by collecting the pellet. The resulting product is an aminated CNF, herein referred to as ACNF-Am. The moisture content of ACNF-Am was determined by drying a known amount of product in an oven at 60 °C for 24 h. The obtained ACNF-Am was kept at 4 °C until further use.

### 4.6. Zwitterionic CNF Formation by Quaternary Ammonium Formation (ACNF-Zwi)

ACNF-Am was solvent exchanged from water to DMSO as described in Section 4.4.1. The DMSO content in the ACNF-Am pellet was determined by drying a known amount of product in an oven at 60 °C for 24 h. The quaternary ammonium reaction proceeded according to a previously reported protocol, with minor adjustments [24]. Briefly, ACNF-Am (0.3 g, dry weight) was suspended in DMSO (10 mL), followed by the addition of SBES (0.18 g). More DMSO was added to decrease the viscosity of the mixture, which was then allowed to react for 24 h at 60 °C. The reaction was terminated by the addition of a large excess of ice-cold DI water. The mixture was washed by centrifugation (4000 rpm, 20 min) and re-dispersion with ice-cold DI water and ethanol 5 times, respectively. The moisture content of the zwitterionic ACNF (ACNF-Zwi) was determined by drying a known amount of product in an oven at 60 °C for 24 h. The obtained ACNF-Zwi was kept at 4 °C until further use.

### 4.7. Characterization

#### 4.7.1. Moisture Content Determination

The moisture content of each modified CNF sample was determined by drying a known amount of sample in an oven at 60 °C for 24 h and recording the weight loss. 

#### 4.7.2. Fourier Transform Infrared (FTIR) Spectroscopy

FTIR spectra of all obtained products were recorded at room temperature using a PerkinElmer Spectrum 2000 FTIR, equipped with an ATR Golden Gate from Graseby Specac (Kent, UK). All spectra were acquired through 64 scans with a spectral resolution of 4 cm^−1^ in the spectral region of 4000–600 cm^−1^. Corrections were made for atmospheric carbon dioxide and water. All spectra were baseline corrected and normalized at the C–O stretch band at 1030 cm^−1^ originating from the cellulose backbone [40]. PerkinElmer Spectrum software was used to analyze all data. 

#### 4.7.3. Zeta (ζ) Potential Measurements

The obtained products were suspended in ice-cold DI water. The concentration of each sample corresponded to 0.3 mg (dry weight)/5 mL. The pH of each sample was in the range of DI water. The samples were immersed in dry ice and then probe sonicated (2 min, pulse on/off for 10 s, respectively) using a CV334 probe sonicator from CIAB Chemical instruments AB with a maximum input power of 750 W, and a maximum frequency of 20,000 times/s at an amplitude of 20%. The samples were then stirred for 30 min before the ζ-potential was measured, using a Zeta potential Zetasizer Nano ZS from Malvern Instruments. Three replicates were carried out for each sample to calculate an average ζ-potential and standard deviation. 

#### 4.7.4. Scanning Electron Microscopy (SEM) and Elemental Analysis via Energy-Dispersive Spectroscopy (EDS)

CNF, ACNF, and ACNF-Zwi were oven-dried overnight at 60 °C. The dried samples were attached to sample supports with double-sided adhesive carbon tape and sputter-coated with a thin layer (~8 nm) of Pt/Pd under an inert atmosphere using a Cressington 208 HR. The samples were then analyzed by ultra-high-resolution field emission scanning electron microscopy (FE-SEM) using a Hitachi S-4800 operating at 5 kV. The elemental composition of each sample was then determined with an X-MaxN from Oxford Instruments. All samples were analyzed at three different regions. For ACNF and ACNF-Zwi, the average weight percent (wt. %) and standard deviation were calculated through the data obtained from these three different areas. 

#### 4.7.5. Acetyl Content Determination

The degree of substitution for acetyl groups in ACNF was quantified using a protocol described elsewhere [41]. Briefly, CNF (100 mg, dry weight) was added to a loosely stoppered bottle containing 40 mL of ethanol (75% v/v). The mixture was stirred for 30 min at 50 °C to allow CNF fibrils to swell. Then, 0.5 N NaOH (40 mL) was measured accurately and added to the mixture, which was left to react for an additional 15 min at 50 °C. The deacetylation was left to proceed at room temperature for 48 h, after which the surplus alkali was titrated with 0.5 N HCl. Phenolphthalein was used as an indicator. The degree of acetyl substitution was calculated according to Equation (2):

(2) $$\frac{{\left(V_{NaOH}\;\left(mL\right)\;\times \;N_{NaOH}\;\left(\frac{mol}{L}\right)\times \;10^{-3}\right)}_{\;consumed\;for\;deacetylation}}{\frac{m_{ACNF}\;\left(g\right)}{\left(M_{Triacetylated\;CNF}\;*\;x\right)\;+\;\left(M_{CNF\;}*\;\left(1-x\right)\right)}}\;=\;3x\left[\frac{mol\;acetyl\;groups}{mol\;ACNF}\right].$$

#### 4.7.6. Carbonyl Content Determination

The degree of substitution of aldehyde groups in ACNF-Ox was quantified using a protocol described elsewhere [42]. ACNF-Ox (100 mg, dry weight) was suspended in DI water (8 mL). The pH of the suspension was adjusted to 4 by 0.1 N HCl. In parallel, 0.25 N hydroxylamine hydrochloride was prepared and the pH was adjusted to 4 by 0.1 N NaOH. The 0.25 N hydroxylamine hydrochloride solution (25 mL) was added to the CNF suspension and the mixture was left to react for 2 h. The mixture was then titrated with 0.1 N NaOH back to pH 4. The degree of aldehyde substitution was calculated according to Equation (3):

(3) $$\frac{(V_{NaOH}\;\left(mL\right)\;\times \;N_{NaOH}\;\left(\frac{mol}{L}\right)\times \;10^{-3}){\;}_{titrant\;consumed}}{\frac{m_{ACNF-Ox\;}\left(g\right)}{\left(M_{ACNF-Ox}\;*\;x\right)\;+\;\left(M_{ACNF\;}*\;\left(1-x\right)\right)}}=x\;\left[\frac{mol\;aldehydes}{mol\;ACNF-Ox}\right].$$

#### 4.7.7. Atomic Force Microscopy (AFM)

The cellulose nanofibers and the modified versions were examined using AFM to understand the morphology of the materials. The samples were prepared by dispersing the freeze-dried nanofibers in water and drop-casting from dilute suspension on mica. An atomic force microscope (AFM) Nanoscope V from Veeco Instruments Inc., Plainview, NY, USA, at a resonance frequency of 310 kHz was used and the nanofiber sizes were evaluated from the height images using Nanoscope V software.

## 5. Conclusions

A sequential modification, including acetylation, periodate oxidation, Schiff base reaction, and borohydride reduction, as well as a quaternary ammonium reaction was devised to prepare zwitterionic acetylated CNF. The presence of the acetyl groups was verified via FTIR spectroscopy, titration, and ζ-potential. The extent of acetylation was determined to 30% via titration, thus mono-acetylated CNF (ACNF) was obtained. The occurrence of aldehyde groups in oxidized ACNF was established via FTIR spectroscopy, titration, and ζ-potential. The extent of oxidation was determined as 34%; a higher degree of oxidation was not desired in order to preserve the fibril structure and crystallinity of the material to a larger extent. The aldehyde groups were used as auxiliaries to introduce secondary amine groups to the structure, as indicated via FTIR spectroscopy and ζ-potential measurements. Lastly, quaternary ammonium cations and sulfonate anions were introduced to the structure. The end product was analyzed via FTIR spectroscopy, ζ-potential, AFM, and SEM-EDS.

## Figures and Tables

**Table 1:** ζ-potential values for CNF and the modified CNF samples prepared by acetylation, oxidation, Schiff base formation, and zwitterionic modification.

Sample	Average ζ-Potential (mV)
CNF	−23.5 ± 1.21
ACNF	−29.8 ± 0.66
ACNF-Ox	−33.7 ± 0.23
ACNF-Am	1.5 ± 1.21
ACNF-Zwi	−6.5 ± 0.17

## Supplementary Materials

The following are available online, Figure S1: SEM images of CNF and zwitterionic acetylated CNF, Figure S2: AFM images of CNF, acetylated CNF and zwitterionic acetylated CNF, Figure S3: EDS spectrum of ACNF-Zwi, Scheme S1: Proposed mechanism for the periodate oxidation of ACNF, Scheme S2: Acid-catalyzed dehydration of the alcohol carbinolamine intermediate in the Schiff base reaction of ACNF-Ox.
