# Supplementary material for: Zwitterionic Acetylated Cellulose Nanofibrils
Source: Molecules. 2019 Aug 29;24(17):3147. doi: 10.3390/molecules24173147 (PMC6749602; doi:10.3390/molecules24173147)
Supplement: Supplementary file 1 [file molecules-24-03147-s001.pdf]

Supplementary information

Zwitterionic Acetylated Cellulose Nanofibrils

Jowan Rostami [1], Aji P. Mathew [2] and Ulrica Edlund [1,*]

[1] Fiber and Polymer Technology, KTH Royal Institute of Technology, SE-10044 Stockholm, Sweden; jowan@kth.se (JR); edlund@kth.se (UE)

[2] Department of Materials and Environmental Chemistry, Stockholm University, Stockholm, Sweden; aji.mathew@mmk.su.se (AM)

* Correspondence: edlund@kth.se

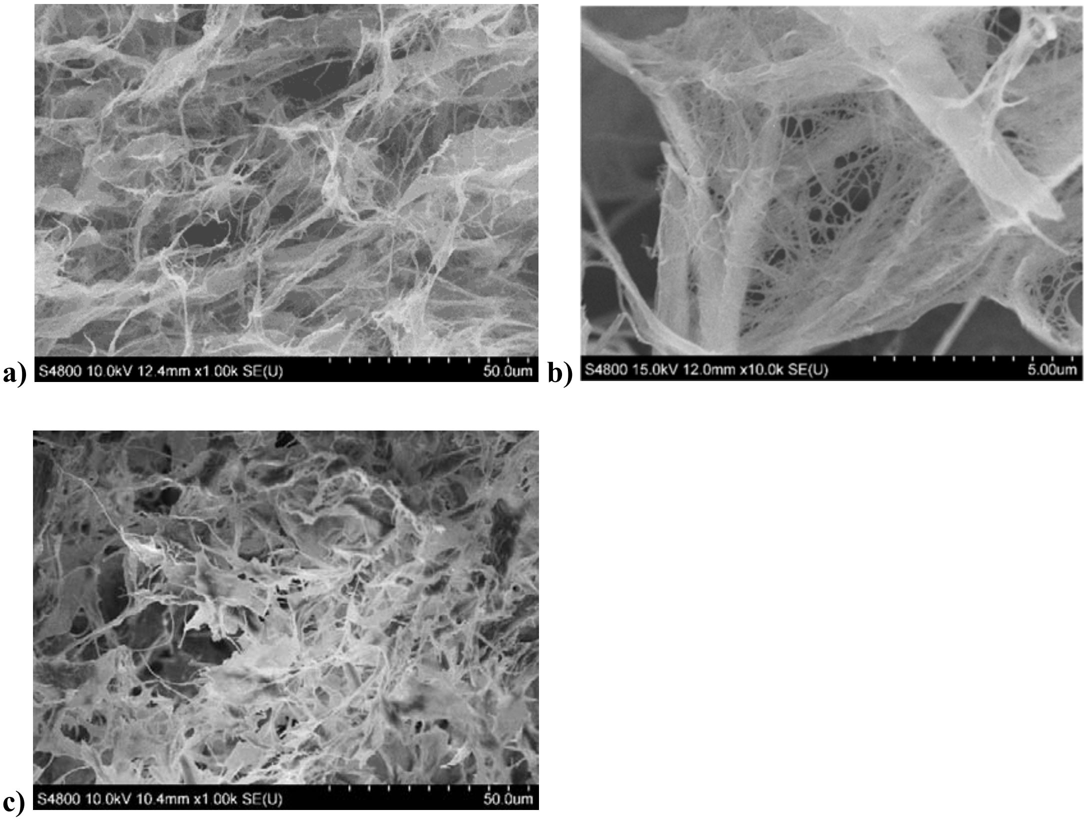

a) b) c)

Figure S1. SEM images of a) CNF with x1000 magnification, b) CNF with x10 000 magnification, and c) ACNF-Zwi with x1000 magnification.

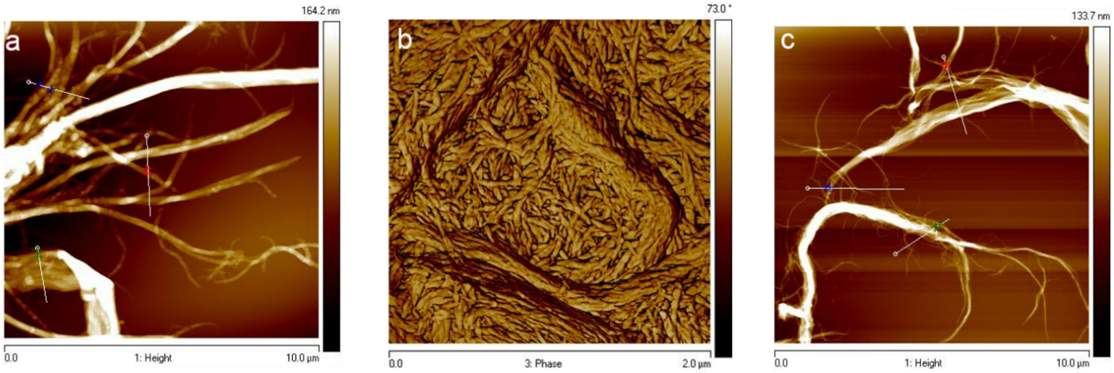

Figure S2. AFM images of a) CNF, b) ACNF, and c) ACNF-Zwi.

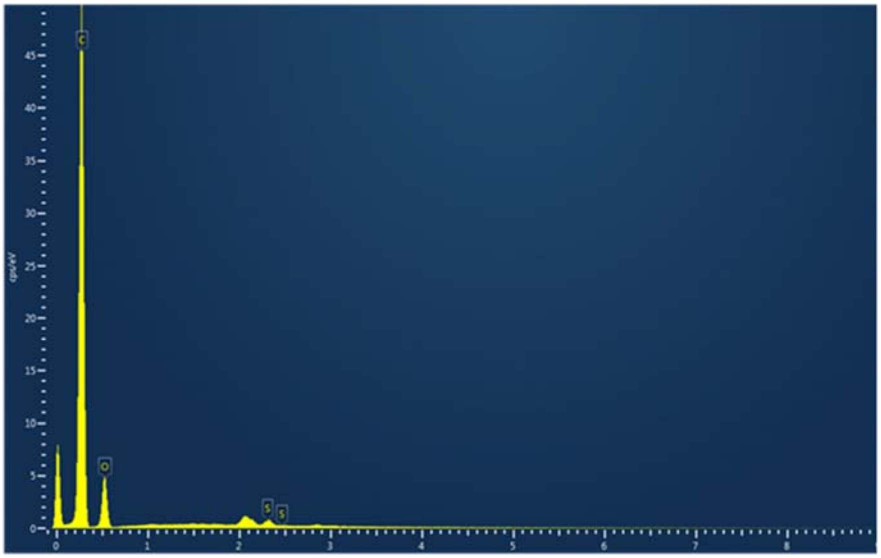

Figure S3. EDS spectrum of ACNF-Zwi.

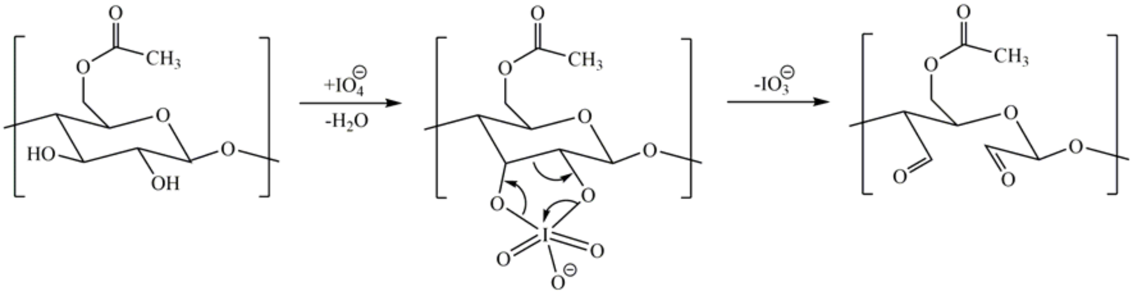

Scheme S1. Proposed mechanism for the periodate oxidation of ACNF.

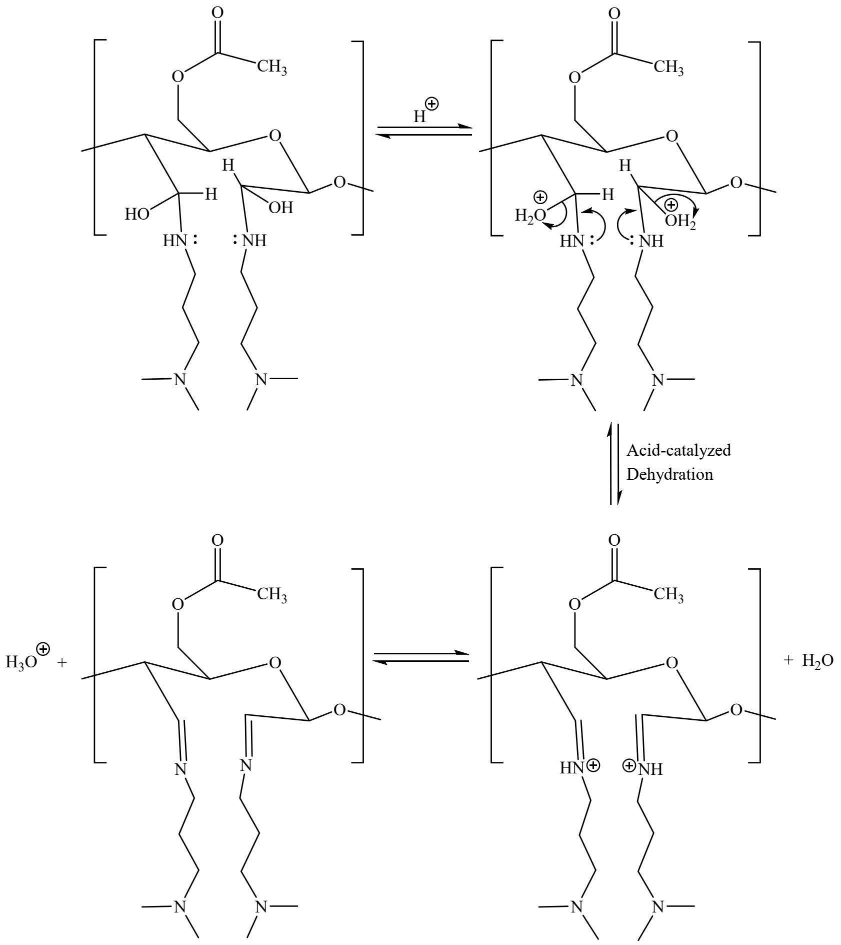

Scheme S2. Acid-catalyzed dehydration of the alcohol carbinolamine intermediate in the Schiff base reaction of ACNF-Ox.
